# Selections

**Published:** 1897-12

**Authors:** 


### Selections.

### CARIOUS TEETH AS A MEANS OF ENTRY INTO THE BODY FOR THE BACILLUS OF TUBERCULOSIS.

Dr. Strarck, of Heidelberg, has reported two cases that clearly show that a decayed tooth may be the point of departure for the invasion of the organism by the bacillus of tuberculosis. In the first case, a young man after a violent toothache had enlargement of the submaxillary lymphatic ganglia on the same side as the affected teeth,—the left lower molars. During the three months following the tumor increased to the size of the fist, painless, irregular of surface, soft and elastic in parts. On the *right* side there was slight enlargement of submaxillary and subclavicular glands, and prolonged expiration in upper lobe of lung. The teeth were extracted, and the tumor, which was in a state of caseous degeneration, was removed. Microscopic examination of the teeth showed numerous bacilli of tuberculosis. The second case, in a girl of fourteen, was very similar. Large numbers of bacilli were found in the extracted tooth. In three cases resembling these two in their clinical aspect, bacilli were demonstrated in the cervical glands, but none could be detected in the carious teeth.

The author believes that not only tubercle bacilli, but also other pathogenic microbes can penetrate the body *via* decayed teeth.

Dental caries appears to be also the most common cause of ordinary chronic lymphadenoma in infants. In one hundred and thirteen cases of infants with enlarged submaxillary ganglia no other cause could be determined in forty-one per cent. of the cases. We see from this the extreme importance of hygiene of the mouth, from the point of view of prophylaxis, during the early years of life.—*Reported in La Revue Médicale, May 23, 1896.*

